# On-site Ultrasound Screens out Asymptomatic Knee Lesions in Elite Adolescent Male Basketball Players

**DOI:** 10.7150/ijms.72299

**Published:** 2022-10-17

**Authors:** Chan-Shien Ho, Tung-Yang Yu, Chien-Hung Chen, Yin-Chou Lin, Wen-Chung Tsai

**Affiliations:** 1 Department of Physical Medicine & Rehabilitation, New Taipei Municipal Tucheng Hospital, New Taipei City, Taiwan.; 2 Center of Comprehensive Sports Medicine, Chang Gung Memorial Hospital, Taoyuan, Taiwan.; 3 Department of Physical Medicine and Rehabilitation, Chang Gung Memorial Hospital, Linkou, Taiwan.; 4 College of Medicine, Chang Gung University, Taoyuan, Taiwan.; 5 Department of Physical Medicine & Rehabilitation, Chang Gung Memorial Hospital, Taoyuan, Taiwan.; 6 Open University of Kaohsiung, Center for General Education, Kaohsiung, Taiwan.

**Keywords:** basketball, injury, ultrasonography, elite, adolescent, patellar tendon, effusion, prevention

## Abstract

Basketball is a popular sport worldwide with a high injury risk. In this study, we conducted survey composed of clinical symptom reporting scale, physical examination and meticulous portable musculoskeletal ultrasound to 19 elite male high school basketball players and 15 regular male high school students. Our study showed the incidence of ultrasonographic findings of any lesion, suprapatellar effusion and proximal patellar tendinopathy is significantly higher in player group, and the incidence of asymptomatic ultrasonographic lesion is also higher in player group. Screening for asymptomatic lesions bares clinical relevance and plays a role in prevention of symptom development. With the concise and easy-to-perform ultrasonography protocol we performed and being interpreted by sports team physician, the protocol can offer precise diagnosis of common injury and screening for asymptomatic lesion potentially progressive.

## Introduction

Basketball is a popular sport worldwide, but also it is a sport with high injury risk. According to a previous report, the incidence of injury is 24.7 events per 1000 playing-hours, a higher incidence compared to other sports 1. It may be due to the character of high intensity and high chance of body-contact between players. The lower limb movements of offensive players include frequent accelerating, decelerating, changing in direction, cutting, jumping and body contact. On the other hand, defensive players also exert to do the movements mentioned above, and even more lateral movement is needed to play defense. High strength, agility, speed and power are required to successfully doing these movements, and they exert a high stress to lower extremities of basketball players 2. According to previous research done to high school varsity basketball players, ankle is the most frequently injured body parts and followed by knee in both male and female student players 3, 4. Although warm up activities might reduce general lower-extremity injuries in basketball players 5 and review has pointed out the importance and effectiveness of preventive programs for anterior cruciate ligament injury (ACL) prevention 6, more study is still needed to formulate injury prevention programs for basketball players to prevent other knee injuries 7. There are several common knee disorders other than ACL injury met by adolescent athletes, such as patellar tendinopathy 8, Osgood-Schlatter disease (OSD), quadriceps tendinopathy 9, iliotibial band (ITB) syndrome 10, medial collateral ligament, lateral collateral ligament and meniscus 11.

Non-invasive screening such as physical examinations, anthropometric indices and balance testing were incorporated to screen for possible risk factors for lower limb injury 12, but still there is no a single best method. Ultrasonography is a mature clinical tool for real-time imaging soft tissue such as muscle, tendon, ligament and even peripheral nerve and robust research has proven its validity compared with golden standard of diagnosis 13-16. When it comes to sideline sports medicine, soft tissue ultrasonography bares advantages of high safety, non-radiative, highly portable, relatively cheap in price and the ability to allow dynamic testing at the field. For many common knee disorders mentioned above, research has shown good sensitivity and excellent specificity of ultrasonographic diagnosis confirmed with magnetic resonance imaging (MRI) 17. Although recent study demonstrated excellent precision of ultrasonography as compared to MRI in the diagnosis of ACL and PCL tears and serve as immediate diagnosis in patients with acute knee trauma 18, we did not include ACL/PCL screening in our protocol since these injury is less likely to be “unknown” because of the injury almost always occur in an acute scenario. The results of previous study showed that, as part of a precareer screening tool, ultrasonography can provide valuable information for determining relative risk of noncontact injuries diagnosed during a 5-year playing career in collegiate American football players in the setting of a prospective longitudinal study 19. A cross sectional comprehensive ultrasonography scanning can not only act a screening for potential injury, but also establish baseline for follow-up study.

By incorporating physical examination, symptom-related scoring scale and knee ultrasonography protocol, we investigated a team of elite high school male basketball player and age-matched control group to delineate the picture of knee injury in this specific studied group.

## Materials and Methods

We enrolled two groups of high-school male students. For the high injury risk group, 19 elite high-school male basketball league players in Taiwan were enrolled, and 15 high school male students with no regular basketball training and no history of sports injury of knee were enrolled as control group. We viewed bilateral knee of a single subject as two independent knees, thus all quantitative data is recorded for both knees. Data collection was completed between September 2019 and August 2021. The study protocol was approved by the institutional review board of Chang-Gung Memorial Hospital, Linkou, Taiwan (#201901232B0) and written, informed consent was obtained from all participating subjects.

### Basic data of subjects

The age, height, body weight, formal training time weekly and time trained as basketball player were recorded.

### Symptoms of knee

For evaluation for symptoms of knee, we employed International Knee Documentation Committee Subjective Knee Form (IKDC-SKF) published by the International Knee Documentation Committee (IKDC) 20. IKDC was established in 1987, and the aim of its establishment is to develop a standardized international documentation system for knee surgery. The “IKDC-Standard Knee Evaluation Form” was published in 1993, and later in 2000, the “IKDC-subjective knee form” (IKDC-SKF) was published. The IKDC-SKF is used in this study due to its property of patient-reported outcome that measures patient's subjective perception of symptoms, function, and symptom-related sports activity and being a knee-specific, rather than disease-specific. The IKDC-SKF questionnaire is composed of 18 items and the possible item score sums up to 87 points. A fully-scored IKDC-SKF represents no impairment and a high participation level perceived by the subject 21. The questions on IKDC-SKF were asked by one of the authors (C.S. Ho) for every enrolled subjects.

### Physical Examination of knee

The physical examination conducted included passive range of motion (PROM) with range of flexion and extension measured with goniometry to screening for any ROM loss. The physical examination was conducted by one of the authors (C.S. Ho).

### Ultrasound examination of knee

We employed qualitative and quantitative methods of ultrasonography for knee examination. The BenQ T3300 portable ultrasound equipped with BenQ L154B (4-15 MHz) linear array probe was used in this study. We examined three views of knee (anterior, medial, lateral) and screened for effusion, ligaments sprain, tendinopathy, bony lesions or any possible lesions generally followed previous report 18. Also, the thickness of distal quadriceps tendon, proximal and distal patellar tendon, distal iliotibial band at insertion to Gerdy's tubercle of tibia and medial collateral ligament at medial joint line were acquired for analysis. (For detail description of ultrasonography examination protocol, please refer to supplementary information.) The ultrasonography examination of knee was performed by all the authors (W.C. Tsai, T.Y. Yu, C.H. Chen and C.S. Ho) following examination protocol, and the performers were blinded to the result of IKDC-SKF and physical examinations. The ultrasonographic images were exported after examinations were done and measurement of thickness was conducted by one of the authors (C.S. Ho), who was blinded to the result of IKDC-SKF and physical examinations during the measuring process.

### Statistics analysis

The scores of IKDC-SKF score between the player and control group, and the subgroup analysis for groups with positive and negative ultrasonography examination findings were compared by unpaired t-test. The results of physical examination between player and control groups were analyzed by unpaired t-test. The incidence of positive physical examination and the incidence of positive ultrasonography lesions between player and control groups were analyzed by Fisher's exact test. The thickness of measured structures of basketball player group and control group was compared with unpaired t-test. A *P*<0.05 is considered statistically significant.

## Results

We interviewed 19 male high-school basketball players and 15 male non-basketball player high-school students and completed physical examination, IKDC-SKF and ultrasonographic screening. Subject characteristics are presented in **Table 1**. The height of player group is significantly higher than control group (180.89±6.42 cm vs 171.51±5.97 cm, *P*<0.01). Also, the time trained as basketball player (4.47±2.14 years vs 0.00±0.00 years, *P*<0.01) and the time of weekly training (33.16±4.48 hours/week vs 0.00±0.00 hours/week, *P*<0.01) are significantly higher in player group. The age (17.37±0.76 years vs 17.67±1.18 years, *P*=0.38) and bodyweight (68.47±5.60 kg vs 69.01±13.34 kg *P*=0.87) showed no difference in the two surveyed groups. The result of physical examination and scores of IKDC-SKF is shown in **Table 2**. The physical examination revealed that passive knee flexion range of motion is higher in player group (125.90±10.35 degrees vs 115.6±10.52 degrees, *P*<0.01), with no difference in passive extension range of motion (0.00±0.00 degree vs 0.20±0.76 degree *P*=0.16). Scores of IKDC-SKF are significantly lower in player group compared with control group in both total scale (77.29±14.20 vs 86.13±2.65, *P*<0.01) and the subscale with sports-related questions (37.74±4.38 vs 39.80±0.81, *P*=0.01).

**Figure 1:**
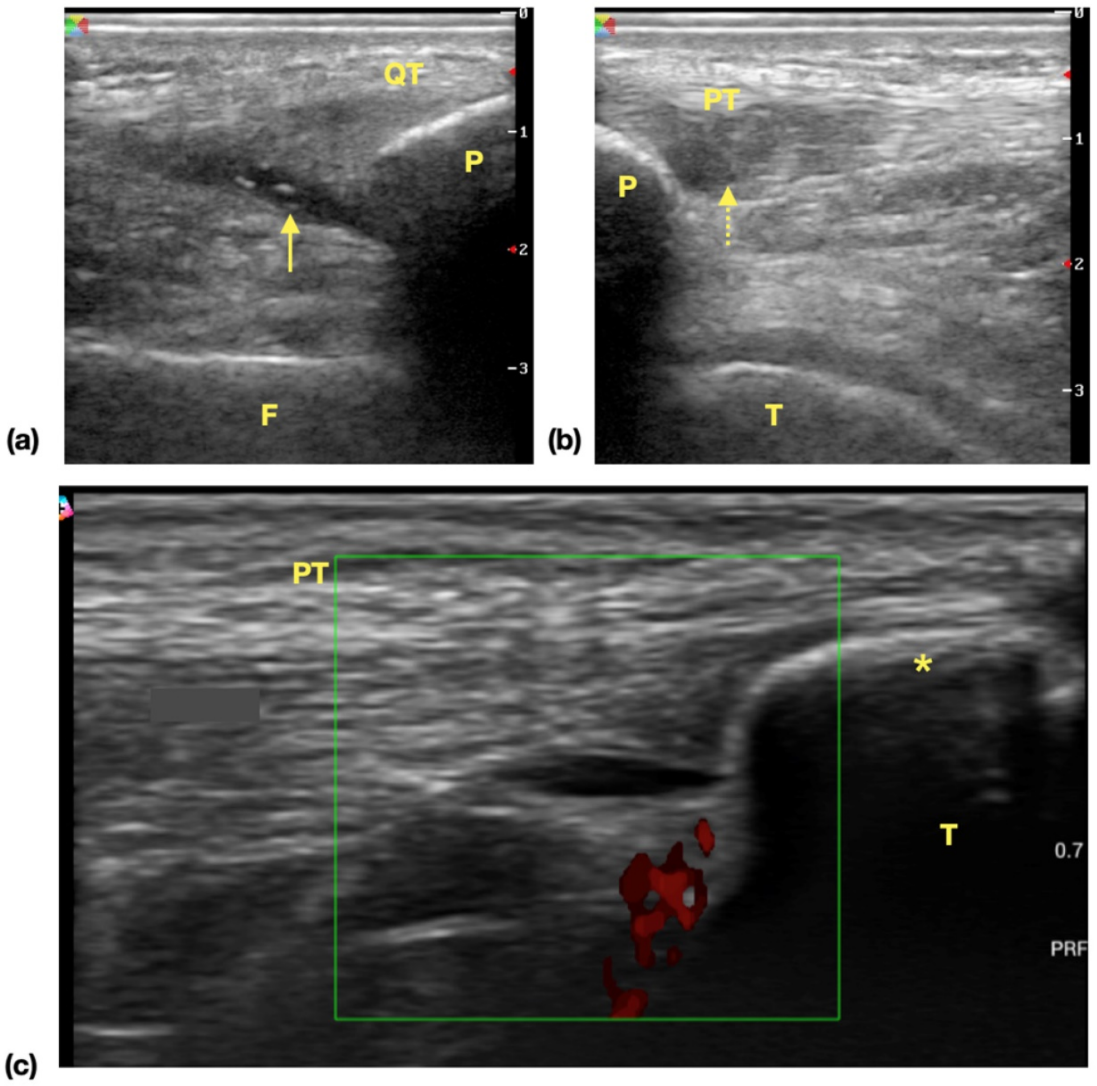
** Common ultrasonographic findings of knee in surveyed athletes. (a)** Effusion of suprapatellar recess. Hypoechoic area (arrow) is noticed in the extent of suprapatellar recess and the findings is suggestive of suprapatellar recess effusion. **(b)** Patellar tendinopathy. Focal hypoechoic change and swelling of proximal tendon is noticed. The normal fibrillar pattern of echogenicity is disrupted (dotted arrow) (c) Tibial tuberosity apophysitis. A separated bone fragment (asterisk) apart from tibia bone is noticed, and power doppler shows increased signals, which is suggestive of inflammatory process. **Abbreviations:** QT: quadriceps tendon, F: femur, P: patella, arrow: effusion, PT: patellar tendon, T: tibia.

The result of ultrasonography examination (**Table 3**) showed significantly higher incidence of at least one positive findings among player groups compared with control group [28 (73.68%) vs 2 (6.67%), *P*<0.01] and also the incidence of at least one ultrasonographic finding among all screened areas [40/266 (15.04%) vs 2/210 (0.95%), *P*<0.01]. In all identified lesions in ultrasonography, the incidence of suprapatellar recess effusion [21 (55.26%) vs 0 (0.00%) *P*<0.01] and patellar tendon lesions [6 (15.79%) vs 0 (0.00%), *P*=0.03] are higher in player groups (Examples of ultrasonographic findings were shown in **Figure 1**). There was no difference in the incidence of medial collateral ligament lesions [6 (15.79%) vs 1 (3.33%), *P*=0.12], tibial tuberosity apophysitis [3 (7.89%) vs 1 (3.33%), *P*=0.62], lesions at iliotibial band insertion [2 (5.26%) vs 0 (0.00%), *P=*0.50] or distal quadriceps tendon lesions [2 (5.26%) vs 0 (0.00%), *P=*0.50]. There was no evidence of lateral collateral ligament injury in both the players and control group subjects. We also found out that in players and control group subjects, the incidence of positive ultrasonography lesion is significantly higher [68% (13/19) vs 4% (1/25), *P*<0.01] in player group. For the subgroup analysis of player group, the scores of IKDC-SKF with positive (n=26) and negative ultrasonography examination findings were compared and the result showed no difference between these groups (IKDC-SKF score 75.31±2.94 vs 81.58±3.39, *P*=0.21).

The measurements of thickness for five structures of knee in ultrasonography (**Table 4**) showed significant difference in thickness of distal patellar tendons [4.16±0.72 mm vs 3.52±0.53 mm, *P*<0.01] and distal iliotibial bands [3.16±0.67 mm vs 3.53±0.58 mm, *P*=0.02] between player group and control group, with no difference in thickness of distal quadriceps tendons [5.30±1.02 vs 5.36±0.81, *P*=0.77], proximal patellar tendons [3.78±1.20 vs 3.88±0.75,* P*=0.70] and medial collateral ligaments [3.01±0.65 vs 2.76±0.69, *P*=0.09].

## Discussion

There are several common knee disorders met by adolescent athlete. Patellar tendinopathy has been shown to be related to jumping sports due to the repetitive stress of the extensor mechanism 8, and its prevalence is considered to be higher in male youth athlete 22. Osgood-Schlatter disease (OSD) is a painful disorder as a structural answer to repeated biomechanical stress due to acute and/or chronic overload to the tibial tuberosity apophysis in adolescent athlete, and it affects mainly adolescent boys active in football and basketball players 23. Quadriceps tendinopathy may result from repetitive high-intensity knee extension especially during high force-generating movement such as jumping. If not properly addressed, tendinosis might progress to partial tear or even complete rupture in extreme circumstances 9. Iliotibial band (ITB) syndrome is a very common cause of lateral knee pain in runners 10, although less commonly met, ITB syndrome can also disturb basketball players. Other injury such as medial collateral ligament, lateral collateral ligament, meniscus and chondral injury were also reported and their rates vary across gender and levels of competition 11.

In previous study, collegiate basketball players with asymptomatic knees screened with MRI showed an incidence of 74% having at least one abnormal finding. Most common abnormalities included bone marrow edema (41%), articular cartilage abnormality (41%), joint effusion (35%), and patellar tendon signal abnormality (24%) 24. Height of the players is an effective screening tools for general knee injury mentioned in one review 12, however, subgroup analysis of injured and uninjured group players failed to show this result. Although the incidence of injury and height are both higher in players group, this might be biased due to the fact that basketball players are apparently taller than general population.

For specific ultrasonographic findings, the incidence of suprapatellar recess effusion was 55.26% and significantly higher than in control group (0.00%). Although ultrasonographic findings, including suprapatellar effusion, popliteal cyst, pes anserinus bursitis, suprapatellar tendinitis, were shown to be significantly correlated with pain intensity in knee osteoarthritis patients 25, no relationship between symptom and knee effusion alone was reported. Additional analysis of our data was done among player groups, which showed no difference of IKDC-SKF score between ultrasonographic effusion positive and negative knees of players (77.82 vs 76.56, P=0.77, unpaired t-test). Also, in a longitudinal follow-up study of collegiate American football players by Lewis et al., there was no higher likelihood for developing knee injury in a 5-year career duration with effusion noted on pre-career ultrasonography screening 19.

The incidence of patellar tendinopathy of player group was 15.79% (6 knees) but only one knee was reported to be symptomatic (IKDC score 49), which indicated that most patellar tendinopathy revealed by ultrasonography in this group is asymptomatic. Fazekas et al. surveyed 31 collegiate athletes involved in jumping movement, 13 of them were male and 9 knees were tested positive of ultrasonographic findings of patellar tendinopathy, but the correlation of positive image findings with the scoring of Victorian Institute of Sport Assessment Questionnaire for Patellar Tendinopathy (VISA-P) was poor and insignificant 26. Our investigation also showed similar result. In previous study of Cook et al., in teenager basketball player aged 14-18 years, if asymptomatic patellar tendinosis is noticed in ultrasonography, there is 30% chance that symptoms will develop in the future, which is significantly higher compared with those who have normal ultrasonographic appearance (9%) 27 and Lewis et al. also revealed that there was a significantly higher likelihood of patellar tendon injury based on the presence of patellar tendon ultrasonographic pathology and the odds ratio was 11 19. These findings made screening of asymptomatic patellar tendinopathy clinically relevant due to the impact on basketball player when it becomes symptomatic. Study also showed that symptomatic patellar tendinopathy is influential to normal foot and ankle alignment, ankle mobility, ITB mobility, hip internal and external rotatory mobility and hip abductor strength 28. For MCL injury, in a previous survey of American high school athletes, there were 11 out of 144 (7.64%) investigated male basketball players having MCL injury 11. The MCL injury was defined positive when athletic trainer reported at the filed or registered in injury reporting system by medical faculties in this study. In our survey, the incidence of ultrasonographic MCL lesion is not significantly higher (15.79%), of which two (5.36%) were reported to be symptomatic. The incidence of tibial tuberosity apophysitis (Osgood-Schlatter disease, OSD) is 7.89%, a higher incidence compared with the work of Foss et al. In their study of American high school basketball players, 4.1% among 122 tested subjects had OSD. A huge difference was noticed that Sinding-Larsen-Johansson Syndrome, which was not found in our survey, was reported to be 3.7% in their survey. A Japanese study showed that the incidence of OSD has a trend of yearly increase during 10 to 14 years old in teenaged male athletes, and the incidence is higher after the secondary ossification center is mature 29. The incidence of OSD is not high, and although it is a self-limiting disease, it can still be prevented by balancing the strength of quadricep and hamstring muscles and adequate stretching. Also, early recognition with proper resting, anti-inflammation measures and physical therapy can lead to good outcome 30. These suggest that screening for OSD is clinically relevant when it comes to high school athletes. There was no distal QT pathology revealed in our studied subjects. However, the screening of QT lesions is still clinically relevant. Lewis et al. showed that there was a significantly higher likelihood of quadriceps musculotendinous injury based on the presence of QT ultrasonographic pathology and the odd ratio were shown to be 140 19.

There was a tested player reporting relatively low IKDC-SKF score with 49 out of 87 and no definite ultrasonographic abnormality was revealed under our ultrasonography examination protocol. This might be explained by that the subject could have knee disorder that cannot be well-detected by ultrasonography 18. Meniscal injury can be revealed in ultrasonography by noticing visible tear, defined as linear hypoechoic deficit along meniscus, or indirect signs such as parameniscal cyst and massive effusion 31. The diagnostic value of ultrasonography on meniscal injury increased due to the improvement in ultrasonographic resolution with high-frequency probe. A review suggests that ultrasonography with a high-frequency linear probe is an acceptable imaging modality in evaluating meniscal tears 32, however, there is still some pitfalls in evaluation of meniscus by ultrasonography, such as poorer accuracy of diagnostic values of lateral meniscus injury than medial meniscus 33. Another reason that correlation of IKDC-SKR with ultrasonography findings was inferior in this tested subject might be not including cartilage scanning in our ultrasonography protocol. Lesions in the patellofemoral cartilage were more commonly observed than those in the femorotibial joint and elevated intrameniscal signal was documented in 20% of subjects examined by MRI, chiefly in the medial meniscus 24. It is reasonable since patellofemoral lesion and deep meniscal lesion are difficult to detect using ultrasonography. Novel techniques taking knee articular motion into consideration was shown to be more valid to significantly decrease false-negative diagnoses compared with fixed-angle transverse scanning 34 and it can be considered to be incorporated in future work. Also in previous study, ultrasonography has a very poor sensitivity for diagnosing patellar cartilage defect, which was reported to be 0 17 and this pathology can also cause symptoms of knee derangement 35 and affect sports activity. These might explain the inferior correlation of IKDC-SKR with ultrasonography findings in this tested subject. On the other hand, we found that in player and control group subjects, the incidence of positive ultrasonography lesion is significantly higher in the player group. It has clinical relevance that in those elite high school basketball players, asymptomatic lesion could be identified via on-site ultrasonography easily and addressed earlier to prevent symptoms development, and decrease the negative impact on training and competition.

There are some limitations of our study. First, although ultrasonography is proved to be reliable in diagnosing and screening for knee injuries, ultrasonography examination is still an operator-dependent image modality. The clinical value of ultrasonography data is based on good image quality and interpretation. Our study was designed to standardize the ultrasonography protocol, and further investigation of intra- and inter-operator reliability of this protocol is needed. Also, the diagnostic value might also vary when different ultrasonography machines are used. We apply portable ultrasonography examination modality so as to serve these players on-site, and the sensitivity of screening can be influenced by the limitation of image resolution using portable machines, and further study can be conducted to compare the difference. Second, the athletes tested positive of ultrasonographic findings were not further examined with other modality such as MRI or direct operation vision. Since conservative or ultrasonography-guided injection therapy is effective in these pathologies, advanced image modality and surgery is not done in the studied group. Last but not least, relatively small sample size in both player and control groups were included and larger sample size examined with the same protocol might be needed to give a more robust result.

In conclusion, following an easy-to-perform, brief and precise on-site meticulous musculoskeletal ultrasonography screening protocol, our study showed similar incidence of common knee injury to previous survey conducted to larger cohort. The protocol can offer preliminary impression of common knee injury the athlete encounter and screening for asymptomatic lesion, which ultimately can benefit the welfare of elite male high school basketball players. Future work can be focused on the longitudinal follow-up of our studied cohort and try to establish predictive value of findings in current proposal.

## Perspective

The findings we shown suggest that on-site ultrasonography can be used to not only diagnose knee lesion but also screen for asymptomatic knee lesion. Our investigation showed potential impact on symptom and injury prevention since we found that in asymptomatic subjects, the incidence of positive ultrasonographic lesion is significantly higher in the player group. It has clinical relevance that in those elite high school basketball players, asymptomatic lesion could be identified via on-site ultrasonography easily and addressed earlier to prevent symptoms development, and decrease the negative impact on training and competition.

## Supplementary Material

Supplementary figures and information.

## Figures and Tables

**Table 1:** Characteristics of high-school basketball players and non-basketball players (N=34)

	Player group	Control group	*P*
Subjects (Number)	19	15	
Age (year-old)	17.37±0.76	17.67±1.18	0.38
Height (centimeter)	180.89±6.42	171.51±5.97	<0.01***
Bodyweight (kilogram)	68.47±5.60	69.01±13.34	0.87
Time trained as basketball player (years)	4.47±2.14	0.00±0.00	<0.01***
Formal training time (hours/week)	33.16±4.48	0.00±0.00	<0.01***

*P<0.05, ** P<0.01, ***P<0.001, unpaired t-test.

**Table 2:** The result of physical examination and IKDC-SKF score

		Player group	Control group	*P*
Knee (number)		38	30	
Knee flexion (degree)		125.90±10.35	115.6±10.52	<0.01***
Knee extension (degree)		0.00±0.00	0.20±0.76	0.16
IKDC-SKF	Total score^a^ (Max.= 87)	77.29±14.20	86.13±2.65	<0.01**
	Sports subscale^b^ (Max.= 40)	37.74±4.38	39.80±0.81	0.01*

^a^The total score of the IKDC-SKF is the original item score from the sum of all items in IKDC-SKF. ^b^The sport subscale is composed of question #8 and #9, which are sports activities related questions located in the page 2 of IKDC-SKF and the sum of all items is 40. Max.: Maximum, *P<0.05, ** P<0.01, ***P<0.001, unpaired t-test.

**Table 3:** Findings of knee ultrasonography

	Player group	Control group	*P*
Knee(number)	38	30	
Number of subjects presented with any ultrasonographic finding	28 (73.68%)	2 (6.67%)	<0.01***
Numbers of ultrasonographic findings in all screened structure†	40/266(15.04%)	2/210(0.95%)	<0.01***
Suprapatellar recess effusion	21 (55.26%)	0 (0.00%)	<0.01***
Patellar tendon lesions	6 (15.79%)	0 (0.00%)	0.03*
Medial collateral ligament lesions	6 (15.79%)	1 (3.33%)	0.12
Tibial tuberosity apophysitis (Osgood-Schlatter Disease)	3 (7.89%)	1 (3.33%)	0.62
Lesions at iliotibial band insertion	2 (5.26%)	0 (0.00%)	0.50
Distal quadriceps tendon lesions	2 (5.26%)	0 (0.00%)	0.50
Lateral collateral ligament lesions	0 (0.00%)	0 (0.00%)	-

†Seven areas were screened for potential lesions. *P<0.05, ** P<0.01, ***P<0.001, Fisher's exact test.

**Table 4:** Thickness of measured knee structures under ultrasonography

	Player group	Control group	*P*
Knee (number)	38	30	
Distal quadriceps tendon (mm)	5.30±1.02	5.36±0.81	0.77
Proximal patellar tendon (mm)	3.78±1.20	3.88±0.75	0.70
Distal patellar tendon (mm)	4.16±0.72	3.52±0.53	<0.01***
Distal iliotibial band (mm)	3.16±0.67	3.53±0.58	0.02*
Medial collateral ligament (mm)	3.01±0.65	2.76±0.69	0.09

*P<0.05, ** P<0.01, ***P<0.001, unpaired t-test.
