# Supplementary material for: On-site Ultrasound Screens out Asymptomatic Knee Lesions in Elite Adolescent Male Basketball Players
Source: Int J Med Sci. 2022 Oct 17;19(13):1856–63. doi: 10.7150/ijms.72299 (PMC9682505; doi:10.7150/ijms.72299)
Supplement: Supplementary file 1 — Supplementary figures and information. [file ijmsv19p1856s1.pdf]

Supplementary Information

Protocol of Ultrasound Examination of Knee in This study

The figures shown bellowed are intended to demonstrate the ultrasound protocol, and these are not pictures of on-site US examinations done while collecting data of this study.

Anterior knee

The subject lies in rest with his knee in full extension. The suprapatellar recess is checked for possible effusion or any fluid accumulation (Sup_figure1). A Finding suggesting suprapatellar effusion can be seen with US as an anechoic collection located inside the recess [1]. Distal quadriceps tendon (QT) is examined with the subject fully flexing his knee. Quadriceps tendinopathy is described as "ill-defined hypoechoic areas located either in the middle or on the sides of the quadriceps tendon" [1]. Color Doppler images is also checked for a hyper-vascular pattern overlying the intratendinous focal hypoechoic areas in QT. Calcifying enthesopathy of QT refers to calcified deposits in the distal portion of QT, and it is defined as irregularly hyperechoic lesions typically with noticeable acoustic shadowing if large enough, and located within the superficial or intermediate tendinous layers of QT [1]. Sono-palpation with the US probe inducing "the pain" can also help confirming the diagnosis. Discontinuity of all the QT layers is checked for any tears of the quadriceps tendon.

After QT is checked, the knee flexion is adjusted to 90 degree and the US probe is further moved to below the patella bone for examination of the whole course of patellar tendon (PT) (Sup_figure 2). In our study, patellar tendinosis and any form of patellar tendinopathy were collectively called "patellar tendon lesion" and counted

as one entity. Patellar tendinosis is defined as hypoechoic area involving the tendon and it can be associated with focal hyperechoic spots and posterior attenuation of the US beam [1]. The loss of normal fibrillar pattern of echogenicity of the PT is also described. Sometimes, a small intratendinous calcification can also be seen within the focal hypoechoic area of PT. Patellar tendinopathy involves the tendon diffusely, and generalized hypoechogenicity and disrupted normal fibrillar pattern of PT can be seen [1]. Traction apophysitis affecting tibial tuberosity (Osgood-Schlatter disease) and the lower pole of the patella (Sinding-Larssen-Johansson disease) can be revealed in US as "…small calcified fragments and irregularities in the bony outlines related to osteochondrosis, focal hypoechoic swelling of the physeal cartilage, hypoechoic degenerative changes in the patellar tendon…." [1]. Any focal hyperemia is also checked with Doppler imaging.

Lateral knee

The subject lies in rest with his knee in internal rotation position and a few degrees less than full extension (Sup_figure 3). The distal segment of iliotibial band (ITB) was first examined to the insertion onto the Gerdy's tubercle, a tubercle located at the anterolateral aspect of the tibial epiphysis. Distal iliotibial band tendinopathy is recognized with US signs of a swollen band characterized by echo texture abnormalities such as hypoechoic changes and loss of the normal fibrillar pattern [1]. Sono-palpation with the US probe inducing "the pain" may help to confirm the diagnosis.

The US probe is then moved to the fibular head to examine lateral collateral ligament (LCL) (Sup_figure 4). LCL sprains and tears appear as a thickened ligament structure with hypoechoic and disrupted fibrillar pattern of ligament [1].

Medial knee

The subject lies in rest with his knee in external rotation position and slight flexion (Sup_figure 5). The medial collateral ligament (MCL) is examined with the probe positioned at the medial knee joint longitudinal to the ligament across the joint. MCL injuries can be revealed by US as a thickened and heterogeneous appearance of the ligament, and a partial tear ligament is recognized based on noticing an irregular hypoechoic component of the ligament with the unaffected part retaining a normal appearance. The Pellegrini-Stieda lesion is formation of a calcification at the femoral insertion of the superficial ligament healed from previous injury, and it can be revealed by US as calcification at the proximal insertion of the MCL [1]. Complete tear of MCL is associated with marked instability and easily recognized as total disruption of the substance of MCL, often associated with joint effusion, meniscal injury and soft tissue lesions.

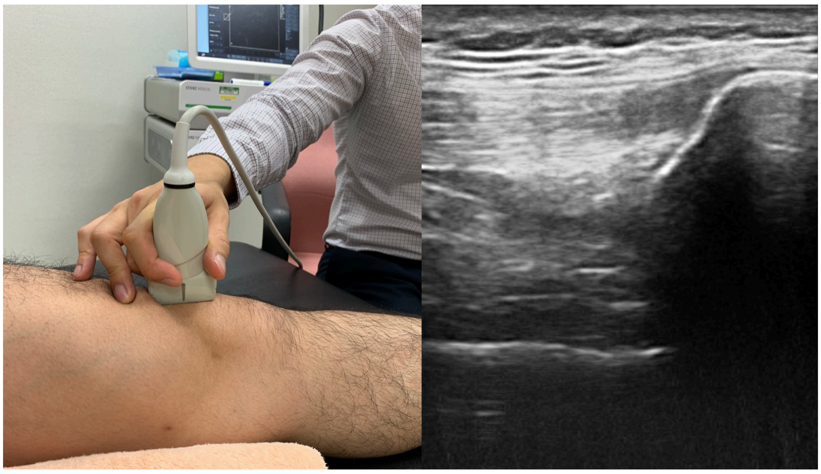

Sup_figure 1. The suprapatellar recess and quadriceps tendon.

The subject lies in rest with his knee in full extension. The suprapatellar recess is checked for possible effusion or any fluid accumulation. Please refer to the text for further information.

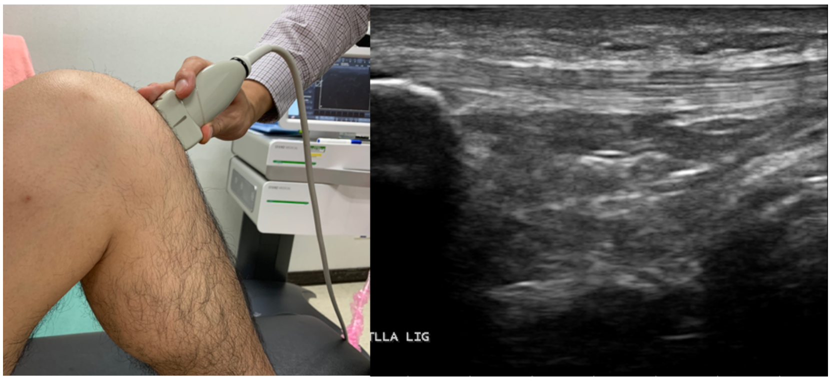

Sup_figure 2. The patellar tendon

The subject lies in rest with his knee in 90 degree flexion. The patellar tendon is examined from its proximal to distal part. Please refer to the text for further information.

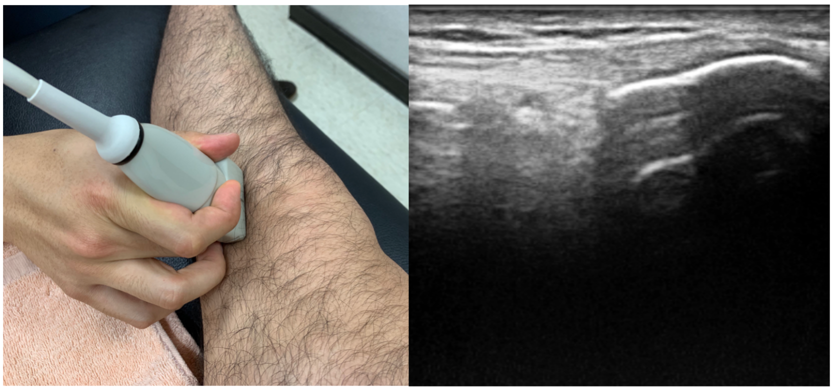

Sup_figure 3. The distal segment of iliotibial band.

The subject lies in rest with his knee in internal rotation position and a few degrees less than full extension. The iliotibial band can be noticed as it inserts onto the Gerdy's tubercle. Please refer to the text for further information.

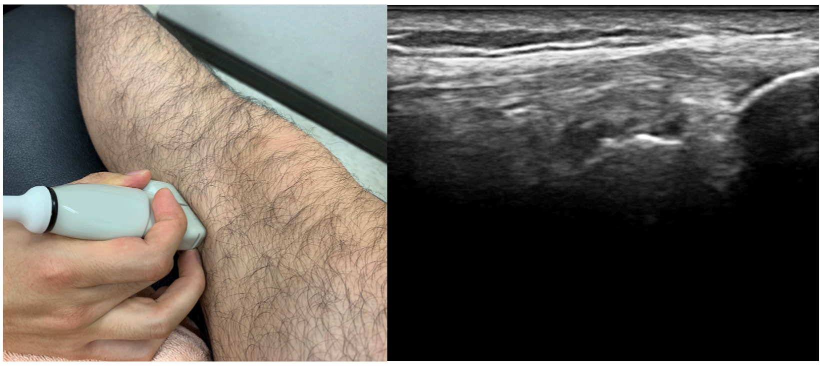

Sup_figure 4. The distal segment of lateral collateral ligament

The subject lies in rest with his knee in internal rotation position and a few degrees less than full extension. The US probe is moved to the fibular head to examine lateral collateral ligament. Please refer to the text for further information.

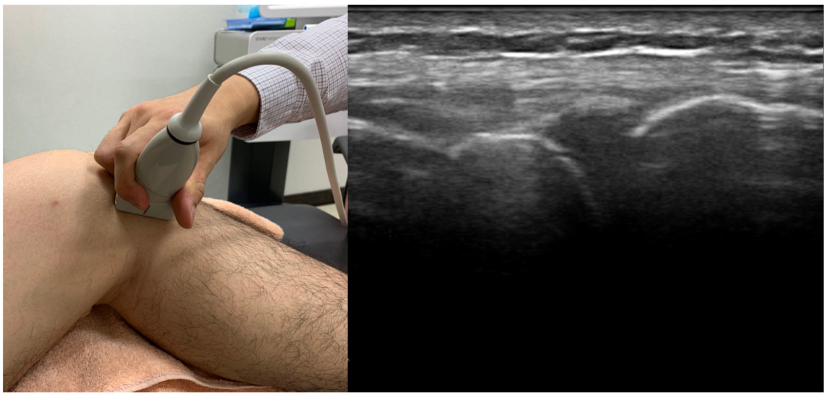

Sup_figure 5. The medial collateral ligament.

The subject lies in rest with his knee in external rotation position and slight flexion. The medial collateral ligament is examined with the probe positioned at the medial knee joint longitudinal to the ligament across the joint. Please refer to the text for further information.

Reference

1. Stefano Bianchi, C.M., *Ultrasound of the Musculoskeletal System*. 1 ed. 2007: Springer Berlin, Heidelberg. XIV, 978.
